# Scrawling

**DOI:** 10.1007/s11606-025-10003-z

**Published:** 2025-11-14

**Authors:** Megan E. Mensinger

**Affiliations:** University of Minnesota Medical School, Minneapolis, MN USA

“Can you prep a note for __?” asks the rounding nurse. “He’s circling the drain.”

I turn to the glowing pixels of my laptop. My fingers compress the keys as I begin the death summary. I only caught a glimpse of the man as I waited outside of the room due to COVID protocols. This is my second death summary as an inpatient medical scribe in training. The first was a woman with a white tube snaking out of her mouth, starkly unnatural plastic against flesh. My ears had just enough time to pick out the words *brain dead* before we were on to our next patient. Later that day, after the family agreed to organ donation, she was wheeled to the operating room and an organ procurement surgery was performed.

I do not remember either of their names now, even though I tried at the beginning. I really did but more than names slipped from my grasp.

I started my scribe job with the hubris of someone who had not yet seen our healthcare system up close. Twenty-two years young, fresh out of college, full of professors’ promises that I was somebody. I was tasked with being the physician’s shadow, typing furiously on a laptop to write their notes, so they could focus on the patient. Like many medical school hopefuls, I needed clinical hours. But I was also captivated by the writing of physicians like Oliver Sacks and Atul Gawande. I wanted to witness the intersection of morality and mortality they wrote about. Armed only with their words and undergraduate pre-med courses, I was woefully unprepared for the humbling, heartbreaking, and haunting experience.

As a scribe, I rounded with the hospitalists on hundreds of patients, observing but not really understanding the body’s delicate balance and what happens when it goes awry. Without any curriculum, I slowly unraveled tell-tale signs. Jaundiced skin and swollen abdomens announced failing livers. Pleasant confusion when asked the year revealed dementia. Black, tarry stools suggested an upper gastrointestinal bleed. My informal self-schooling was one of witnessing, pouring over notes, Googling, and asking the physicians questions. At this tertiary care center, I was always steps behind. My mind swirled with lab results and acronyms to decode as I slammed into the limits of what I could teach myself, and I documented an endless scrawl of details I did not understand.

There to do a job, I was not a student. Few people cared what I saw or what I learned, and there were no formal mentors to turn to. Mundanity obscured the extraordinary circumstances of my day. Clock in, clock out. It is easier to just keep moving. Dwell and you drown. But me, I get stuck on things. I am a furious fixator, and the tragedies I was witnessing began to seep through.

- A woman flies past me on a stretcher, vomiting thick burgundy blood into a previously transparent bag. The rounding nurse, and I stare, with wide eyes. The doctor mutters “likely esophageal varices.” A thought emerges in my mind. My grandfather died of those. And then we continue with our day.
- A man’s cries reverberate through me as he receives an HIV diagnosis as a death sentence. And as he is breaking, a physician who did not live through the early 1980s, tries their best to explain there are treatments now. But stigma is its own kind of deadly.
- Everyone rushes past me into the room of a patient who was alert and conversational twenty minutes ago. Resuscitation protocols begin. The physician calls the patient’s wife. She is on her way but will not arrive fast enough. The next call is the time of death.

Countless people, and I know almost nothing of their lives. But I was witness to some of their most vulnerable moments. While preparing the charts, I would often see imaging results and diagnoses before the patient knew about them. After reading countless radiologist reports, phrases such as “concerning for metastases” would fill me with dread. And then I would watch as the physician delivered the difficult news. A 22 year-old kid with no training in patient care, standing in the corner. My tidal blue eyes silently bearing witness. Not speaking unless spoken to and offering nothing for the pain.

***

It is the season of the snow. In the distance, the wide banks of the Mississippi bask. The elevator spits me out on an inpatient cardiac floor, a couple of stories into the sky. I am 27 years old now, in my second semester of medical school, and about to see my first patient as a medical student. My current curriculum provides the context I have been craving, and there are mentors to guide me through the labyrinth of medicine.

After my tribulations as a scribe, I almost walked away from medicine for good. But with my hand on the door I hesitated. All those hours and all that knowledge gained; all the lives wheeled past me. I owed myself the opportunity to do better than bear witness.

I take a deep breath as I walk into my patient’s room. She is an elderly woman with a warm smile who is gracious as I stumble through my questions.

*"How long have you been feeling short of breath*?"

*"What medications do you take*?"

I do my best to gather her story, medical and beyond. I will not squander my first opportunity to make a connection with a patient. I will know something of her life, lived much longer than my own, and my hippocampi will hold onto this moment.

There is so much left to learn in my training. I find it daunting and beautiful as I ponder the rites of passage left for me to tread. But I also feel an undercurrent that has eluded me since I graduated college. A faint pulse of peace, getting stronger. A settling in my soul.

My potential, a promise that might just be fulfilled.

A chance to offer something for the pain.

